# The addition of S100B to guidelines for management of mild head injury is potentially cost saving

**DOI:** 10.1186/s12883-016-0723-z

**Published:** 2016-10-20

**Authors:** Olga Calcagnile, Anders Anell, Johan Undén

**Affiliations:** 1 Department of Paediatric Medicine, Halmstad Regional Hospital, Halmstad, Sweden; 2 Barnkliniken, Hallandssjukhus Halmstad, 301 85 Halmstad, Sweden; 3 Institute for economic research, Lund university school of economics and management, Lund, Sweden; 4 Department of Anaesthesiology and Intensive Care, Skåne University Hospital, Malmö, Sweden

## Abstract

**Background:**

Mild traumatic brain injury (TBI) is associated with substantial costs due to over-triage of patients to computed tomography (CT) scanning, despite validated decision rules. Serum biomarker S100B has shown promise for safely omitting CT scans but the economic impact from clinical use has never been reported. In 2007, S100B was adapted into the existing Scandinavian management guidelines in Halmstad, Sweden, in an attempt to reduce CT scans and save costs.

**Methods:**

Consecutive adult patients with mild TBI (GCS 14-15, loss of consciousness and/or amnesia), managed with the aid of S100B, were prospectively included in this study. Patients were followed up after 3 months with a standardized questionnaire. Theoretical and actual cost differences were calculated.

**Results:**

Seven hundred twenty-six patients were included and 29 (4.7 %) showed traumatic abnormalities on CT. No further significant intracranial complications were discovered on follow-up. Two hundred twenty-nine patients (27 %) had normal S100B levels and 497 patients (73 %) showed elevated S100B levels. Over-triage occurred in 73 patients (32 %) and under-triage occurred in 39 patients (7 %). No significant intracranial complications were missed. The introduction of S100B could save 71 € per patient if guidelines were strictly followed. As compliance to the guidelines was not perfect, the actual cost saving was 39 € per patient.

**Conclusion:**

Adding S100B to existing guidelines for mild TBI seems to reduce CT usage and costs, especially if guideline compliance could be increased.

## Background

Head injury is a serious health problem in developed countries and associated with a substantial economic burden [1]. Most (up to 95 %) of head injuries are classified as mild head injury (MHI), commonly defined as Glasgow Coma Scale (GCS) 13-15 with the presence of certain risk factors such as loss of consciousness (LOC) and/or amnesia [2, 3].

Typical management of MHI involves computed tomography (CT) of the brain to identify complications such as intracranial haemorrhage and cerebral contusions [4]. These complications are rare but may occasionally need neurosurgical intervention [5]. Guidelines have therefore recommended liberal CT examinations in this patient group. Patients with GCS 15 and no risk factors have a very low risk of intracranial complication [6] and can be discharged from the emergency department (ED) without a CT scan [7].

Due to the considerable resource use and high number of unnecessary CT scans, recent efforts have been concentrated on optimizing CT use after MHI [7–12]. These decision rules are based upon risk factors from patient history and clinical examination. However, due to the high socioeconomic cost of missing cases of intracranial complication, CT rates remain high [12].

Another aspect to be taken into account is the logistics of patients waiting in the ED to have a CT scan. Some departments may obtain a CT result within minutes but in smaller facilities patients may need to wait several hours before a CT can be carried out, stocking the work flow at the ED [12].

Several groups have considered the use of brain biomarker S100B in this clinical setting. Studies show that serum levels of the protein may reduce CT scans in the MHI subgroup of patients by 30 % without missing intracranial complications [13–15]. Serum levels of S100B are also not affected by ethanol intoxication [16] and represent an objective addition to the more subjective risk factors included in existing guidelines. Despite theoretical reports of the potential of S100B to reduce costs in this patient group, no reports of clinical S100B use, and hence actual cost and time reduction, exist.

In the year 2000, the Scandinavian Neurotrauma Committee (SNC) published guidelines for management of non-severe head injuries [7]. In 2007, serum S100B measurements were introduced into these guidelines at Halmstad Regional Hospital, Sweden, based upon the evidence available at the time, in an attempt to reduce CT scans, costs and waiting time in the ED. The aim of the present study was to establish if this change in management routines resulted in a decrease in health care costs and waiting time for patients.

## Methods

### Study setting and population

**Fig. 1:**
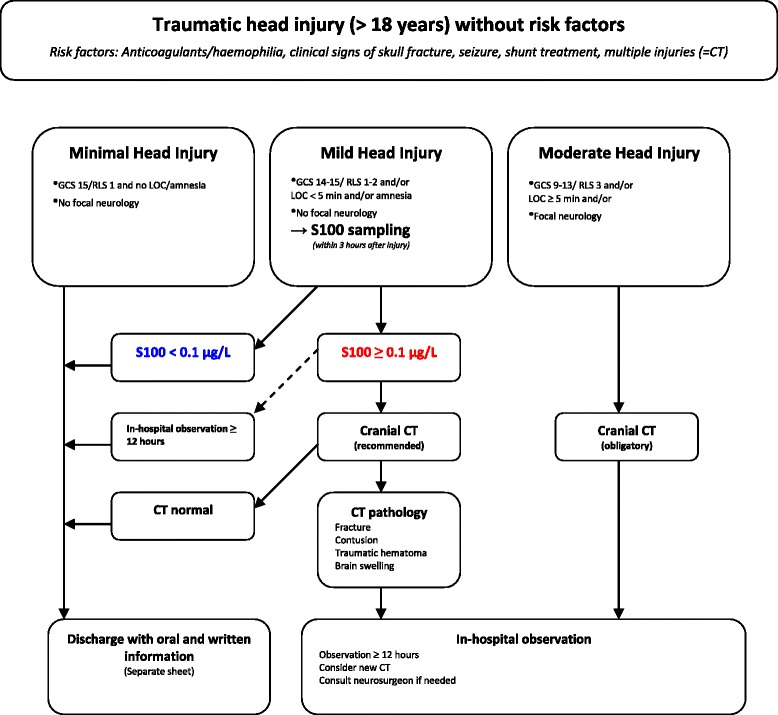
modified Scandinavia Neurotrauma Committee guidelines including S100B sampling

The study setting is the Halmstad Regional hospital, Sweden; a level II trauma centre with 24-h emergency care, anaesthesiology, radiology, surgery and intensive care. From November 2007 (6 months following the introduction of S100B into clinical care, see Fig. 1 for management routines) to December 2013 we prospectively enrolled consecutive adult patients with MHI and S100B sampling, according to these clinical guidelines. In September 2013, an update to the Scandinavian Guidelines was introduced. This study did not take into account the new guidelines and age or antiplatelet medications were not considered as risk factors. The inclusion criteria in this study were: adult patients with acute trauma to the head with GCS 14-15 during examination and/or loss of consciousness for less than 5 min with no neurological deficits nor additional risk factors (therapeutic anticoagulation or haemophilia, clinical signs of depressed skull fracture or skull base fracture, posttraumatic seizures, shunt-treated hydrocephalus and multiple injuries). According to SNC guidelines, trauma history was not considered as a risk factor.

Exclusion criteria were: age less than 18 years, non-Swedish citizens (difficult to follow up) and patients where serum sampling for S100B was done more than 3 h post-injury.

Ethical approval was granted from the regional ethics board (approval number 19/2007).

### S100B analysis

A 5 ml blood sample was drawn from patient’s cubital vein in the ED. Samples were analysed with the fully automated Elecsys® S100 (Roche AB) at the Clinical Chemistry Department of Halmstad Regional hospital, Sweden, with results being available to treating physicians within 1 h. Based on previous studies [13, 14], we set a cut-off level for normal levels of S100B at less than 0.10 μg/L and a window of sampling of within 3 h from the time of the accident.

### CT examinations

CT scans were performed with a GE VCT Ligthspeed 64 multislice CT scanner including 10 mm thick slices. CT scans are always analysed by a board certified radiologist.

### Data registration and follow-up

Details of how patients were managed, including patient characteristics, injury type, patient history, clinical examination results, current medications, CT details including time needed from the writing of the request to the radiologist result, admission type and duration were documented in a pre-determined database.

Compliance to the guidelines was calculated by examining the actual patient management compared to the suggested management from the guidelines. All patients were asked to answer a questionnaire sent by mail 3 months after the injury. The questionnaire was repeated if no answer was received. If no answer was received from these attempts, patients were contacted via telephone. Included in this questionnaire were questions that would identify a significant intracranial lesion [9], occupation, data concerning sick-days, new contacts with medical professionals and information concerning functionality and quality of life. In cases where patients could not be reached by mail or telephone, medical records and national mortality databases were consulted for evidence of complications and/or death. Patients who would suffer significant (enough to seek medical care) intracranial complications after discharge would therefore be identified.

Data was registered on an Excel® file. Descriptive statistics was analysed using IBM SPSS® Statistics Version 20 software. Comparison of number of sick days between the two groups of patients was performed with the non-parametric Mann–Whitney *U*-test.

### Cost analysis

The Swedish health care is state-owned; it is partially difficult to determine the costs on an individual basis considering that state refund of hospital expenses are based on hospital annual budget more than refund per service. Our cost analysis is therefore based upon standard costs according to our hospital accounts or (where data is missing) national reports. The average cost for S100B analysis during the study period was 21€ and the average cost for a non-contrast cranial CT was 130€. The cost of one day in the surgery ward (the typical admission ward for MHI patients) was 600€. Using data from the OCTOPUS study [4], the costs for a patient that is admitted only for MHI observation was calculated to be 61 % of the total costs, i.e. 366€ a day. We decided not to calculate a monetary value regarding the opportunity costs related to time spent by patients in the ED (difficult to assess) and we did not consider socioeconomic costs associated with increased cancer risks from CT scans at all (theoretically based). Not considering these aspects would lead to an under-estimation of the cost-saving potential of S100B implementation.

## Results

We enrolled 795 patients with MHI and S100B levels. Sixty-nine patients were excluded according to exclusion criteria: 15 patients were younger than 18 years of age, 45 patients did not live in Sweden, 9 patients had their S100B blood sampling more than 3 h post-injury. The final population was therefore 726 patients. Descriptive statistics are presented in Table 1.

**Table 1:** Descriptive statistics

	S100B < 0.10 μg/L	S100B ≥ 0.10 μg/L	All
Male	140 (61.1 %)	305 (61.3 %)	445 (61.3 %)
Female	89 (38.9 %)	192 (38.7 %)	281 (38.7 %)
Age (mean)	31, 8 years (Range 18-89y)	46, 6 years (Range 18-92y)	42, 2 years
Alcohol intoxication	94 (41 %)	231 (46.4 %)	325 (44.7 %)
Total	229	497	726

**Fig. 2:**
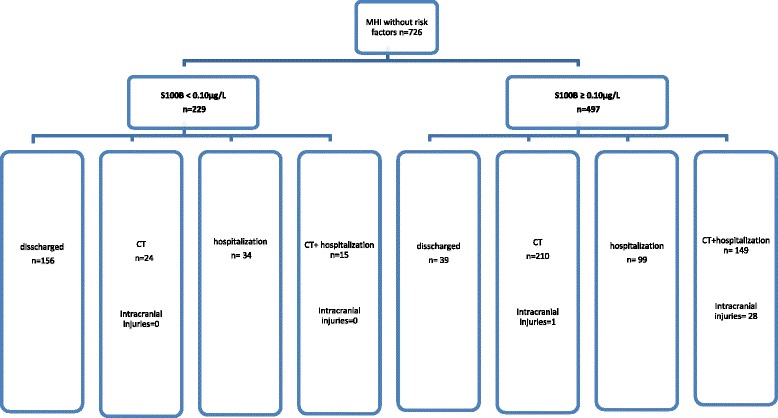
Patients management in the study cohort including number of intracranial injuries. CT = computed tomography; MHI = mild head injury

Compliance to guidelines was reasonable; more than 67 % of patients were managed according to guidelines. Two hundred twenty-nine patients had a S100B lower than 0.10 μg/L and among them 156 patients (68 %) were directly discharged without a CT or being admitted for in-hospital observation (Fig. 2). Even among patients with elevated S100B levels, we registered cases of poor compliance to the guidelines where patients with normal CT were admitted to hospital or patients with normal 12-24 h in hospital observation still underwent a CT scan (121 patients) (Fig. 2).

Thirty- two patients had pathology on CT but only 29 of these (4.7 %) were classed as traumatic abnormalities (isolated skull fracture *n* = 4, cerebral contusions *n* = 9, acute subdural hematoma n = 3, intracranial air *n* = 2, combinations of traumatic intracranial findings *n* = 11). No patients needed neurosurgical intervention. One patient with a small cerebral contusion was dismissed without hospitalization. One patient died as a result of the head injury; an 83-year-old male with expansive cerebral contusions that later resulted in a fatal intracranial pressure increase. He had an admission S100B level of 0.23 μg/L. Details of how patients were managed are presented in Fig. 2.

The follow up questionnaire was completed for 589 patients (81 %), consisting of 190 patients with normal S100B levels (83 % of population with normal S100B levels) and 399 patients with elevated S100B levels (80 % of population with elevated S100B levels). No patient with negative S100B levels sought the emergency room for missed complications. In the questionnaire, patients reported number of sick-days; there was no significant difference in number of sick-days between patients with normal S100B levels and those with elevated levels (*p* = 0.352).

**Fig. 3:**
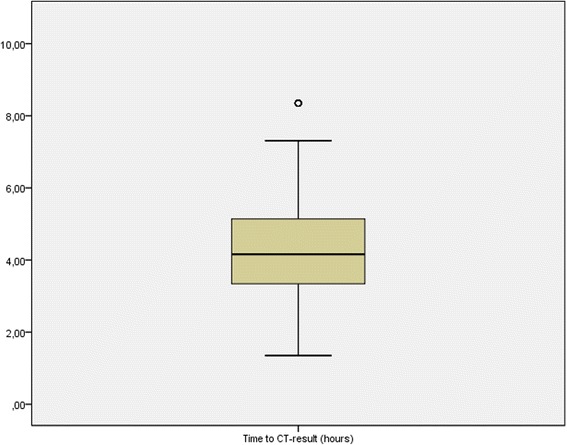
Time to CT-result (hours)

Average waiting time to CT was 4 h and 14 min, calculated from the 398 patients that underwent a CT examination, with a waiting time range from 1 h and 35 min to 8 h and 35 min (Fig. 3).

The actual cost were calculated for the 726 patients strictly taking into account only S100B analysis, CT and hospitalisation cost, for an average of 242 € per patient.

To calculate the potential reduction in cost, we calculated several potential costs given different assumptions (Table 2): 1) potential cost if S100B is not used in the guidelines and assuming the same practices regarding CT and hospitalization for all patients as for the 570 patients that had high S100B levels in the actual cohort (281 € per patient), and 2) potential cost if the guidelines with S100B are followed strictly and assuming that only CT is used, as recommended in the guidelines, for the 497 patients with S100B levels higher than 0.10 ug/L (110 €). If the guidelines were followed strictly and CT only was used as the management option, the potential savings per patient was 71 € for this cohort. Given the actual use of S100B and CT/ hospitalization for our cohort, the calculated savings was limited to 39 € per patient.

**Table 2:** Actual cost for 726 patients = cost for S100B + cost for all the CT taken + cost for all the patients hospitalized

		S100 = 21 €	CT = 130 €	Hospitalization = 366 €	Tot
ACTUAL COST in follow-up (cost per patient)		726 × 21 € = 15 246 €	398 × 130 € = 51 740 €	297 × 366 € = 108 702 €	175 688 € (242 €)
POTENTIAL COST given different assumptions	S100B not in guidelines and assuming same use of CT and hospitalization as for cohort		0,7 x 726 x 130 € = 66 066 €	0,52 x 726 x 366 € = 138 172 €	204 238 € (281€)
	Strict compliance based on guidelines for S100 + CT only	726 × 21 € = 15 246 €	CT (S100B+) 497 × 130€ = 64 610 €		79 856 € (110 €)

Potential cost for 726 patients given different assumptions:

-if S100B is not included in guidelines= 156 patients with S100B negative were directly dismissed, we calculated an hypothetical cost if they underwent a CT or were hospitalized

-strict compliance based on guidelines= we considered that all the patients with a negative S100B were dismissed (138 patients) and took into account only the cost for S100 B positive patients

## Discussion

Considering the scarcity of health care resources, socioeconomic aspects of patient management should be fundamental [12, 17]. MHI is a common reason for ED contact and is associated with considerable use of health care resources [17]. These are partly due to the ineffective triage of patients to either discharge or further examinations/admission. However, these routines have been warranted due to the significant consequences of missing a significant brain injury after MHI for both patients and health care providers [18].

Although several rules have been suggested in MHI management, they are only based upon positive predictors, i.e. risk factors that should lead to a CT scan if present. The decision to incorporate S100B into the existing SNC guidelines in our hospital in 2007 was based upon the negative predictive ability of this biomarker, i.e. an aspect that could potentially reduce resource use. Since 2007, additional studies and a meta-analysis have confirmed findings showing the potential of S100B to safely reduce CT scans in this patient group [19–21].

Our findings show a reduction in costs after S100B implementation in a typical ED setting. However, compliance to the new guidelines regarding S100 and use of CT and hospitalization was not perfect and both over- and under-triage was observed. Since the routines were relatively new (we allowed 6 months before initiating the study) it is understandable that physicians over-triaged patients with normal S100B levels. None of these showed any intracranial complications.

It is important that guidelines in this setting show a very high sensitivity (high negative predictive value) for significant intracranial injuries. The cost of missing a patient with such a complication is substantial [18]. Even though we have included over 700 patients in our study, a much larger cohort would be needed to include enough patients with significant complications to clearly examine this aspect. However, it may be unreasonable to expect 100 % sensitivity in a guideline and clinical advice and/or follow-up should be included in order to identify and treat patients missed from the initial triage [11].

Adapting our results into other cohorts may be difficult. Firstly, adapting S100B into guidelines other than the SNC proposal will naturally show different results. However, independent economic and clinical comparisons of the most prominent decision rules have shown the SNC guidelines to be similar, if not superior, in performance [8, 12, 22]. Despite this, validation and cost analysis of clinical S100B use in other guidelines using other cohorts are naturally warranted. Also, costs for the different aspects of the management routines will differ between sites. Caregivers should, however, be able to adapt their costs into our results to give an estimation of the economic impact our management routines in other health care systems. Finally, our results are based upon some assumptions regarding the use of CT and/or hospitalization. The guidelines recommend CT as the primary management option. However, our results show that many patients were hospitalized, sometimes in addition to CT scanning. Our assumptions therefore also included a model including the use of CT and hospitalisation that was observed in the present cohort.

## Conclusion

Adding S100B to existing guidelines as a negative predictor for normal CT scans is potentially cost saving, although actual savings will ultimately be determined by compliance to guidelines and local costs for CT and hospitalisation.

The biomarker should be considered as a clinical tool, especially when CT rates of MHI patients are high.

## Abbreviations

CT: Computed tomography
ED: Emergency department
GCS: Glasgow Coma Scale
LOC: Loss of consciousness
MHI: Mild head injury
SNC: Scandinavian Neurotrauma Committee
